# Could preoperative and postoperative optimal nutrition support modulate the inflammatory response and clinical outcome of severe malnourished surgical patients with gastrointestinal neoplasia?

**DOI:** 10.1186/cc14476

**Published:** 2015-03-16

**Authors:** L Mirea, D Pavelescu, I Grintescu

**Affiliations:** 1 Emergency Hospital Floreasca, Bucharest, Romania

## Introduction

Our aim was to assess whether perioperative and postoperative optimal 7-day nutrition support could modulate the inflammatory status and clinical outcome of severe malnourished patients with surgery for gastrointestinal neoplasia.

## Methods

A prospective randomized study of 64 patients with gastrointestinal neoplasia, severe malnourished BMI <18.5, albumin level <3 g/dl, BW loss >10%, NRS >3, scheduled for surgery, allocated into two groups. Group A: 32 patients, minimal enteral nutrition in the postoperative period according to tolerance, medium 500 kcal/day. Group B: 32 patients received optimal parenteral nutrition support (25 kcal/kg/day) 3 days before surgery and continued for at least 4 days postoperatively. We measured CRP, fibrinogen, IL-6, TNF, albumin level preoperative and at 96 hours, the incidence of complications, and the length of ICU stay.

## Results

**Figure 1:**
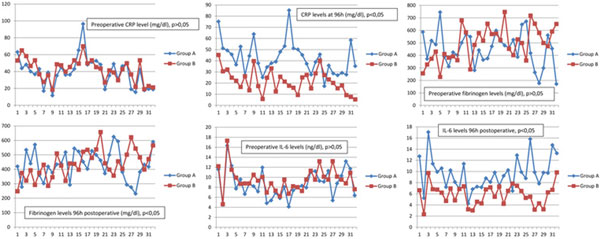
**Results 1**.

**Figure 2:**
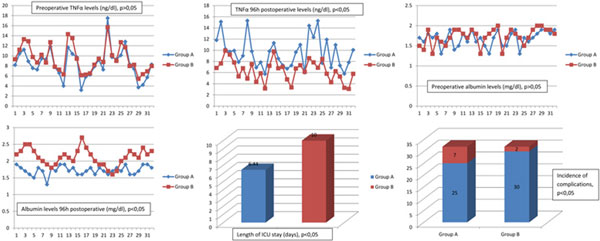
**Results 2**.

There was a significant decrease in the values of CRP, IL-6, TNF, albumin at 96 hours in group B. No difference in fibrinogen. A significantly lower rate of complications and a shorter time of ICU stay were observed in group B. See Figures 1 and 2.

## Conclusion

Perioperative optimal nutrition support for at least 7 days could modulate the inflammatory status and clinical outcome of severe malnourished surgical neoplasic patients.

